# Posterior condylar offset changes and its effect on clinical outcomes after posterior-substituting, fixed-bearing total knee arthroplasty: anterior versus posterior referencing

**DOI:** 10.1186/s43019-019-0022-2

**Published:** 2020-01-01

**Authors:** Moon Jong Chang, Seung-Baik Kang, Chong Bum Chang, Do Hwan Han, Hyung Jun Park, Keummin Hwang, Jisu Park, Il-Ung Hwang, Seung Ah. Lee, Sohee Oh

**Affiliations:** 1 Department of Orthopaedic Surgery, Seoul National University College of Medicine, SMG-SNU Boramae Medical Center, Seoul, 07061 South Korea; 2 Department of Orthopaedic Surgery, Seoul National University College of Medicine, Seoul National University Hospital, Seoul, 03080 South Korea; 3 Department of Emergency Medicine, Seoul National University College of Medicine, Seoul National University Hospital, Seoul, 03080 South Korea; 4 Department of Physical Medicine and Rehabilitation, College of Medicine, Kyung Hee University, Seoul, 05278 South Korea; 5 Department of Biostatistics, SMG-SNU Boramae Medical Center, Seoul, 07061 South Korea

**Keywords:** Anterior referencing system, Posterior referencing system, Posterior condylar offset, Posterior condylar offset ratio

## Abstract

**Background:**

We sought to determine whether there was a difference in the posterior condylar offset (PCO), posterior condylar offset ratio (PCOR) and clinical outcomes following total knee arthroplasty (TKA) with anterior referencing (AR) or posterior referencing (PR) systems. We also assessed whether the PCO and PCOR changes, as well as patient factors were related to range of motion (ROM) in each referencing system.

**Methods:**

This retrospective study included 130 consecutive patients (184 knees) with osteoarthritis who underwent primary posterior cruciate ligament (PCL)-substituting fixed-bearing TKA. The difference between preoperative and postoperative PCO and PCOR values were calculated. Clinical outcomes including ROM and Western Ontario and McMaster University (WOMAC) scores were evaluated. Furthermore, multiple linear regression analysis was performed to determine the factors related to postoperative ROM in each referencing system.

**Results:**

The postoperative PCO was greater in the AR group (28.4 mm) than in the PR group (27.4 mm), whereas the PCO was more consistently preserved in the PR group. The mean postoperative ROM after TKA was greater in the AR group (129°) than in the PR group (122°), whereas improvement in WOMAC score did not differ between the two groups. Preoperative ROM was the only factor related to postoperative ROM in both groups.

**Conclusions:**

There was no difference in postoperative PCO in AR and PR group and the PCO was not associated with postoperative ROM. PCO was more consistently preserved after surgery in the PR group. The postoperative PCO and PCOR changes did not affect the postoperative ROM. Furthermore, similar clinical outcomes were achieved in the AR and PR groups.

**Trial registration:**

Retrospectively registered (Trial registration number: 06-2010-110).

## Background

Proper implant positioning and sizing are crucial for successful total knee arthroplasty (TKA) [1–4]. Anterior referencing (AR) and posterior referencing (PR) systems are the two major systems for positioning and sizing of the femoral component. The AR system has the reduced risk of femoral notching or patellofemoral overstuffing, whereas posterior condylar resection is not set. In contrast, the posterior condylar resection is set in the PR system, whereas the anterior femoral cutting is less predictable [5]. Therefore, each referencing system has its own advantages and disadvantages.

However, in contemporary TKA, traditional concepts of the AR or PR system might not be as certain [5, 6]. Theoretical disadvantages of each referencing system could be overcome by meticulous surgical techniques, advanced surgical instruments and newly developed implants. Some recent implant systems adopt the femoral component that has a smaller mediolateral dimension. In PS knee, because the PCL is removed, the flexion gap is often widened. Thus, if there is increased flexion gap during surgery in the AR system, it could be compensated with by up-sizing the femoral component without mediolateral overhang. In addition, there are implants that have a femoral component with an increased cutting angle of the anterior flange to avoid AFC notching, and thus, down-sizing the femoral component is possible in the PR system.

On the other hand, preservation of the posterior condylar offset (PCO) and posterior condylar offset ratio (PCOR) was reported to be related to the degree of maximal flexion after TKA [7–16]. The PCO or PCOR after TKA could differ based on the referencing system used. Theoretically, the PCO or PCOR can be consistently preserved in the PR system because the same amount of resected bone is replaced by the femoral component. Thus, it is reasonable to speculate that the PR system could lead to greater flexion after TKA. However, besides the PCO and PCOR, there are many factors that could be related to range of motion (ROM) after TKA, such as preoperative ROM, posterior slope of the tibial component and the type of implant used [14, 17, 18]. In addition, the factors related to ROM after surgery could differ between the AR and PR systems. However, there is no consensus that exist regarding these issues [11–13, 19].

Therefore, we sought to determine whether there was a difference in PCO and PCOR after TKA between the AR and PR systems. We also attempted to determine whether PCO and PCOR changes, as well as patient factors, were related to ROM after TKA using each referencing system. In addition, we aimed to examine whether the improvement of clinical outcomes after TKA differed between the two referencing systems. We hypothesized that restoration of the PCO and PCOR after TKA would be better in the PR system than the AR system, and that changes in the PCO and PCOR would be related to ROM after TKA using both referencing systems. Finally, we hypothesized that the clinical outcome after TKA using the PR system would be better than that using AR system.

## Methods

This was a retrospective study that started with 154 patients, who underwent primary TKA using the same prosthesis due to end-stage osteoarthritis (OA) at our institution, from February 2014 to November 2015. Of these, 16 were excluded because of a history or objective evidence of rheumatoid arthritis [5], posttraumatic arthritis [6], concomitant neuronal disease [3], weakness or severe instability in the operated limb [2]. Patients who with less than 2 years of follow-up were also excluded [8]. Consequently, 130 patients (184 knees) with OA who underwent primary TKA were included in this study. There were 118 females (91%) and 12 (9%) males, with a mean age of 72 years old (standard deviation [SD], 6.2 years; range, 57 to 88 years). The mean preoperative height and weight were 152.2 cm (SD, 6.0; range, 138 to 173.9 cm) and 62 kg (SD, 8.7; range, 33 to 84 kg). The mean body mass index (BMI) was 26.7 kg/m2 (SD, 3.4; range, 16.8 to 36.6 kg/m2). The median follow-up was 37 months (range, 26 to 47 months). Patients were categorized into two groups according to the referencing system used. There were no differences in sex, BMI and laterality of the operated limb between the two groups, even though the mean age was higher in the AR group (*p* = 0.008) (Table 1). The study protocol was approved by the institutional review board of authors’ hospital (IRB number: 06–2010-110).

**Table 1:** Comparisons of the patient demographics and preoperative variables between the AR and the PR groups

Variable	AR group	PR group	*p*-value
	(*n* = 93)	(*n* = 91)	
Sex, Female	64 (94%)	54 (87%)	0.550
Age (years)	73 ± 6.1	71 ± 6.0	0.008
BMI (kg/m^2^)	26.5 ± 3.6	26.5 ± 3.6	0.494
PCO (mm)	28.1 ± 3.6	27.4 ± 2.8	0.126
PCOR	0.47 ± 0.04	0.47 ± 0.03	0.394
ROM (°)	120 ± 17.1	120 ± 16.6	0.804
WOMAC	44.2 ± 15.9	38.0 ± 12.6	0.038

Data are presented as the means and standard deviations (SD)

Abbreviations: *AR* anterior referencing, *PR* posterior referencing, *PCO* posterior condylar offset, *PCOR* posterior condylar offset ratio, *ROM* range of motion, *WOMAC* Western Ontario and McMaster University osteoarthritis index, *BMI* body mass index

All surgeries were performed by a single senior surgeon using the same surgical techniques, except for the referencing system (AR or PR system) used. An anterior midline incision and medial parapatellar arthrotomy was performed with a tourniquet applied. The posterior cruciate ligament (PCL) was substituted with a fixed-bearing implant. All knees were implanted with the U2® Total Knee System (United, Taipei, Taiwan). All femoral, tibial, and patellar components were cemented. When using the AR system, the stylus was positioned in the highest position of lateral aspect of anterior femur to determine the anterior cutting level. Anterior femoral cutting was performed to a perfect match for the anterior cortical line. A larger femoral component was chosen to prevent an increased flexion gap while using PCL-substituting implant if the femoral component was measured between two sizes (Table 2). In contrast, when using the PR system, preliminary anterior resection using the cutting zig of the up-sized femoral component was performed to assess the possibility of AFC notching. Then, the down-sized component was selected if possible to prevent overstuffing of patellofemoral joint. We aimed to cut the proximal tibia at a 0-degree slope in all patients.

**Table 2:** Proportion of femoral component size selected in each referencing system

Variable	AR group	PR group
	(*n* = 93)	(*n* = 91)
Size as it is	49 (53%)	71 (78%)
Up-sized	39 (42%)	3 (3%)
Down-sized	5 (5%)	17 (19%)

Data are presented as the numbers of patients, with the proportions in parentheses. Abbreviations: *AR* Anterior referencing, *PR* posterior referencing

**Fig. 1:**
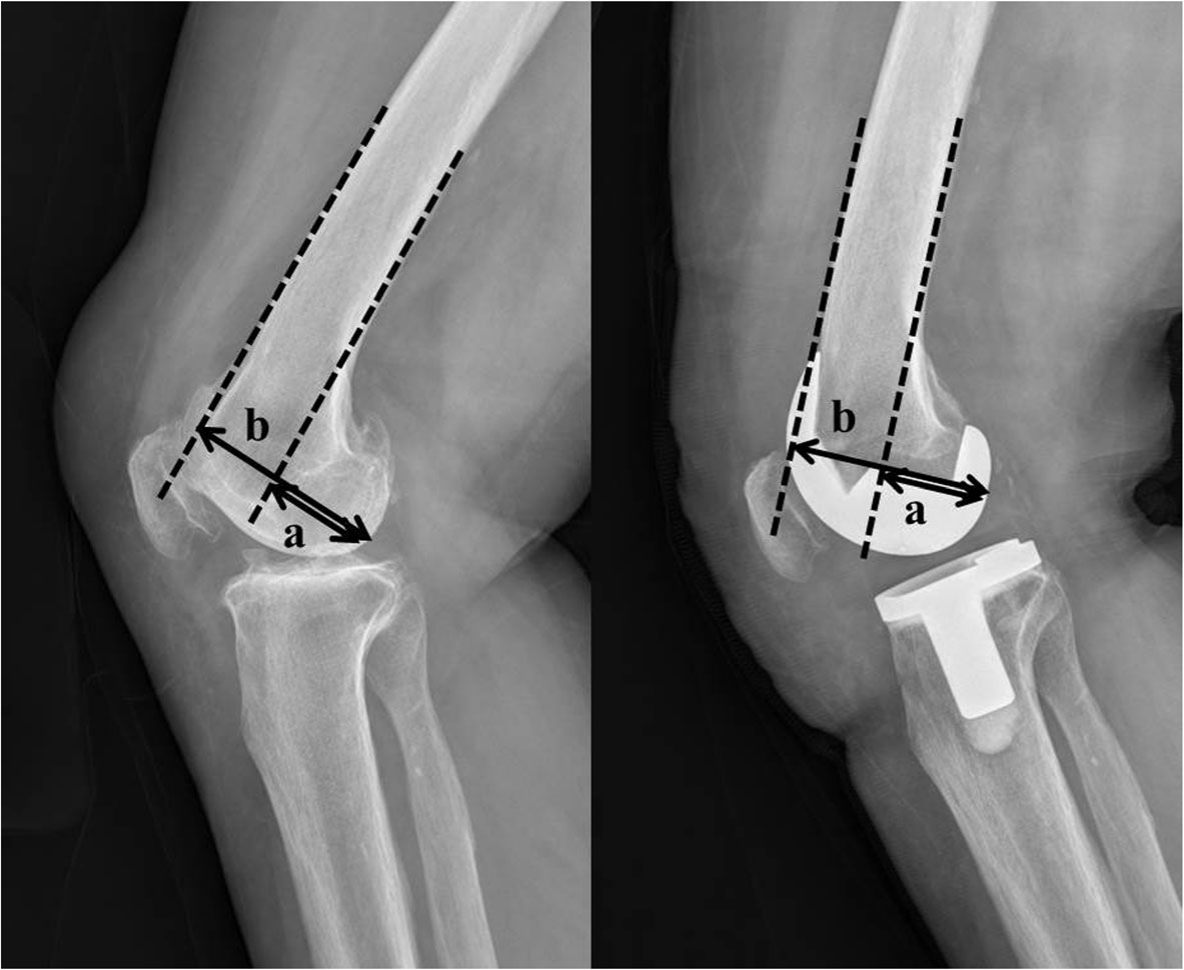
Measurement of the posterior condylar offset (PCO, a) and posterior condylar offset ratio (PCOR, a/b) based on true lateral preoperative and postoperative knee radiographs. **a** distance in millimeters from the tangent of the femoral diaphysis posterior cortex to the posterior condylar margin; **b** distance in millimeters from the posterior condylar border to the tangent of the femoral diaphysis anterior cortex

We performed radiographic evaluations using a picture archiving and communication system (PACS) (MaroviewTM, Marotech, Seoul, Korea). The conventional radiographs taken preoperatively and at the last follow-up visit were used. True pre- and postoperative lateral radiographs, with perfect overlap of the medial and lateral femoral condyles, were used in all patients. The PCO was evaluated on the lateral radiographs by measuring the maximum distance between the tangent of the femoral diaphysis posterior cortex and the posterior condylar margin (Fig. 1) [14]. The PCOR was calculated by dividing the PCO by the maximum distance between the posterior condylar border and the tangent of the femoral diaphysis anterior cortex (Fig. 1) [20]. The preoperative data showed no differences in terms of mean PCO and PCOR between the AR and PR groups (*p* = 0.126 and *p* = 0.398, respectively) (Table 1). Inter- and intra-observer reliabilities for the PCO and PCOR measurements were performed using intra-class correlation coefficients (ICC). The two observers repeated the measurements two times within a 2-week interval. The ICCs of the intra-observer reliabilities (PCO, 0.94; PCOR, 0.86) and inter-observer reliabilities (PCO, 0.89; PCOR, 0.83) were satisfactory.

Clinical outcomes were evaluated by an independent clinical investigator preoperatively and 2 years postoperatively. The presence of a flexion contracture and maximum flexion, in a supine position, were measured using a goniometer. Western Ontario and McMaster University (WOMAC) scores were used to evaluate the clinical outcome after surgery.

Statistical analyses were performed using SPSS for Windows (version 19.0; SPSS Inc., Chicago, IL), and *p*-values of < 0.05 were considered statistically significant. Demographic and clinical data such as age, BMI, PCO, PCOR, ROM and WOMAC score were described as means and standard deviations (SD). Continuous variables between the two groups were compared using the Student’s t-test. The proportions of the categorical variables in the two groups were compared by using the chi-square test. The change between pre- and postoperative values was determined using the paired t -test.

To evaluate how well the PCO and PCOR were preserved in each referencing system, the differences between pre-and postoperative PCO values were classified into 3 groups: group 1 (≤ − 2 mm), group 2 (− 2 mm < PCO difference ≤ 2 mm), and group 3 (> 2 mm). In addition, the differences between the pre-and postoperative PCOR values were also classified into 3 groups: group I (≤ − 0.03), group II (− 0.03 < PCOR difference ≤ 0.03), and group III (> 0.03). The reference value (2 mm) for the PCO difference was derived from a previous study that found the mean cartilage thickness of femur to be 2.2 mm [21]. We assumed that ±2 mm difference of the PCO is an acceptable change considering that a plain radiograph cannot account for the thickness of the cartilage. In addition, *Johal et al*. mentioned that a mean difference of 0.03 for the PCOR was observed after TKA, so we postulated that a PCOR difference of more than 0.03 after surgery was meaningful [22]. To determine the statistical significance of the difference in the proportions of PCO and PCOR changes between the two groups, the Chi-square test was used. In addition, the one-way analysis of variance (ANOVA) was used to determine whether there was a difference in postoperative ROM among the three groups, which was divided by the amount of changes in the PCO and PCOR after surgery. The multiple linear regression analysis using variable with *p* < 0.05 was performed to determine the factors that were related with postoperative ROM. Independent variables included age, BMI, sex, tibial slope and PCO changes and preoperative ROM.

## Results

**Fig. 2:**
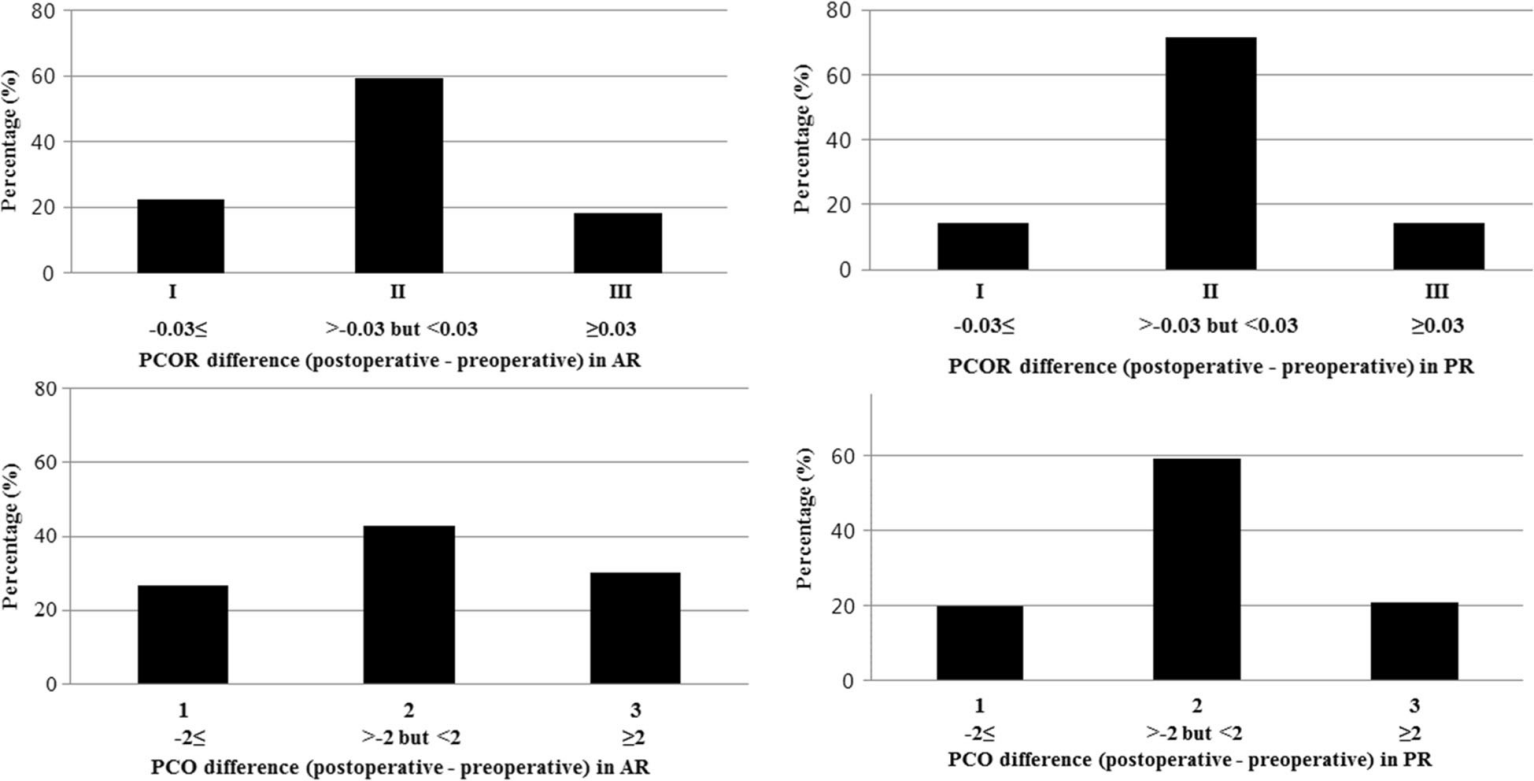
Proportional differences of the knees (%) between pre- and post-TKA based on the posterior condylar offset (PCO) and posterior condylar offset ratio (PCOR). The proportion of the preserved PCO (−2 mm < PCO difference ≤ 2 mm) was higher in the PR group than the AR group (*p* = 0.039)

The postoperative mean PCO value was 28.4 mm in the AR group and 27.4 mm in the PR group (*p* = 0.038), whereas the PCO values were more consistently preserved in the PR group. And there was no difference in the mean postoperative PCOR values between the two groups (*p* = 0.392). In terms of the mean amount of changes in the pre- and post-TKA PCO and PCOR, there was no significant difference between the two groups (*p* = 0.567 and *p* = 0.988, respectively) (Table 3). However, the proportion of the knees with a relatively well preserved PCO (− 2 mm < PCO difference ≤ 2 mm) was higher in the PR group than the AR group (*p* = 0.039) (Fig. 2). And there was no significant notching case like violation of the outer and the inner table of the anterior femoral cortex in down-sizing PR group (*n* = 17).

**Table 3:** Comparisons of the postoperative variables between the AR and the PR groups

Variable	AR group	PR group	*p*-value
	(*n* = 93)	(*n* = 91)	
PCO (mm)	28.4 ± 3.5	27.4 ± 2.8	0.038
PCOR	0.47 ± 0.04	0.47 ± 0.04	0.392
PCO changes (mm)	0.22 ± 3.6	0 ± 2.4	0.567
PCOR changes	0 ± 0.37	0 ± 0.25	0.988
ROM (°)	129 ± 4.6	122 ± 14.3	< 0.001
WOMAC	22.3 ± 9.8	17.1 ± 9.6	0.001

Data are presented as the means and standard deviations (SD)

Abbreviations: *AR* anterior referencing, *PR* posterior referencing, *PCO* posterior condylar offset, *PCOR* posterior condylar offset ratio, *ROM* range of motion, *WOMAC* Western Ontario and McMaster University osteoarthritis index

**Fig. 3:**
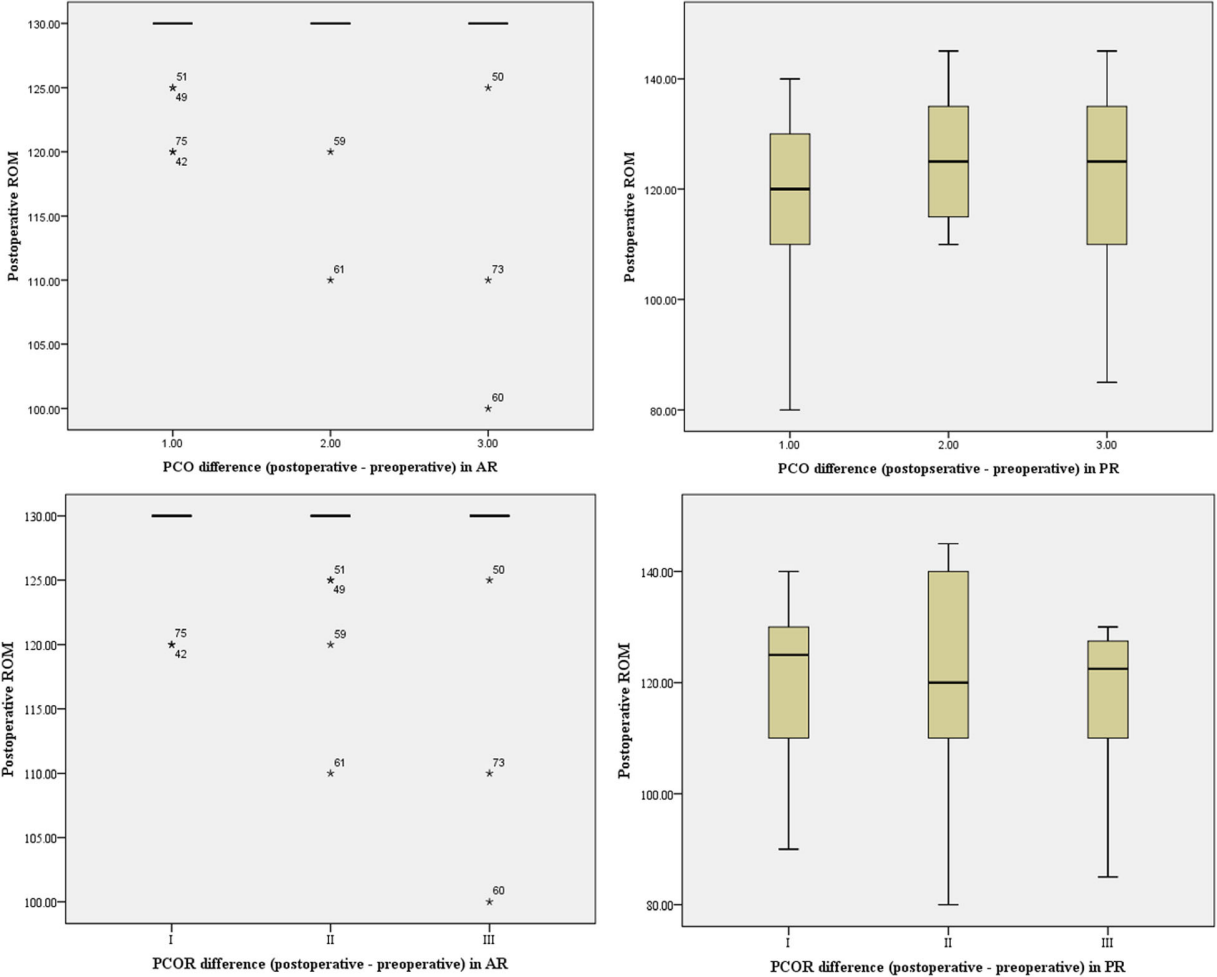
Postoperative range of motion, according to the difference between pre- and post-TKA based on posterior condylar offset (PCO) and posterior condylar offset ratio (PCOR). Postoperative ROM did not differ regardless of PCO and PCOR differences in both the AR (*p* = 0.935 and 0.688) and the PR group (*p* = 0.940 and 0.552)

There was no difference in postoperative ROM, according to the amount of PCO and PCOR changes in the AR group (*p* = 0.935 and 0.688) and the PR group (*p* = 0.940 and 0.552) (Fig. 3). The preoperative ROM was the only factor related to the postoperative ROM in both groups (Tables 4 and 5).

**Table 4:** Factors related to the postoperative range of motion in the AR group

Variable	Univariable analysis		Multivariable analysis	
	β	*p*-value	β	*p*-value
Age	−0.004	0.955		
Sex, Female	0.460	0.814		
BMI	0.052	0.700		
Preoperative ROM	0.078	0.009	0.078	0.009
Tibial slope	0.098	0.654		
PCO changes	−0.184	0.160		
Adjusted R^2^				0.067

Data are presented as the *p*-values and regression coefficients (β)

Abbreviations: *AR* anterior referencing, *BMI* body mass index, *ROM* range of motion, *PCO* posterior condylar offset

**Table 5:** Factors related to the postoperative range of motion in the PR group

Variable	Univariable analysis		Multivariable analysis	
	β	*p*-value	β	*p*-value
Age	− 0.142	0.589		
Sex, Female	−2.500	0.640		
BMI	−0.989	0.045	−0.733	0.123
Preoperative ROM	0.292	0.001	0.269	0.003
Tibial slope	−0.263	0.816		
PCO changes	0.322	0.614		
Adjusted R^2^				0.123

Data are presented as the *p*-values and regression coefficients (β)

Abbreviations: *PR* posterior referencing, *BMI* body mass index, *ROM* range of motion, *PCO* posterior condylar offset

The mean postoperative ROM and its improvement after TKA were greater in the AR group than the PR group, whereas improvement in WOMAC score did not differ between the two groups. The mean postoperative ROM was significantly greater in the AR group (129°) than in the PR group (122°) (*p* < 0.001) (Table 3). Moreover, the proportional changes in ROM after TKA was also significantly higher in the AR group (9°) than in the PR group (2°) (*p* = 0.007). The postoperative mean WOMAC was better in the PR group than in the AR groups (17.1 vs. 22.3, *p* = 0.001) (Table 3). However, the change in WOMAC after TKA showed no difference between the two groups (*p* = 0.205).

## Discussion

The postoperative PCO and PCOR have been shown to be related to postoperative ROM, although results have been controversial [8, 12–14]. Furthermore, the postoperative PCO or PCOR can be affected based on the referencing system used. The principal findings of this study were: 1) There was no difference in postoperative PCO in AR and PR group and the PCO was more consistently preserved in the PR group; 2) changes in PCO and PCOR after surgery were not associated with postoperative ROM in the AR and PR groups, and the only related factor for the postoperative ROM was the preoperative ROM regardless of referencing system used; 3) although the postoperative ROM was better in the AR group, there was no difference in improvement of WOMAC scores after surgery between the AR and PR groups.

Our findings did not completely support the hypothesis that restoration of the PCO and PCOR would be better in the PR group compared to the AR group. The portion of knees with a well-preserved PCO was higher in the PR group. However, the mean postoperative PCO was greater in the AR group. *Almeida et al*. reported that there was no difference in the postoperative PCO and PCOR between the AR and PR groups [7]. In the previous study, the mean postoperative PCO was 27.4 mm in the AR group and 27.7 mm in the PR group (*p* = 0.32). Although the implants used in the previous study were different than those used in this study, the results of the postoperative PCO and POCR values were similar. Furthermore, the previous study compared the medial and lateral PCO values between AR and PR systems using computed tomography, which revealed that the PCO values after TKA were greater in the AR group than in the PR group [11]. This study selected a larger implant for femurs in-between sizes to prevent flexion instability. Therefore, the proportion of up-sized femoral component was higher in the AR group, and this could affect the greater postoperative mean PCO in the AR group (Table 2). Therefore, our findings suggest that the postoperative PCO and PCOR could be preserved in both the AR and PR systems using a contemporary surgical technique and implant.

In the current literature, it is controversial whether the postoperative PCO and PCOR are related to postoperative ROM. Initially, *Bellemans et al*. suggested the concept of the PCO, and they found the relationship between the postoperative PCO and postoperative ROM in TKA performed using a PCL-retaining implant [8]. The previous study explained the cause of ROM limitations using the phenomenon of early impingement between the posterior cortex of the femur and the posterior lip of the polyethylene insert. This finding was caused by several factors, such as a paradoxical roll forward with flexion, a reduced posterior slope of the tibial component and a high posterior lip in the polyethylene insert [12]. *Malviay et al*. showed similar findings in patients with TKA using a PCL-retaining implant, and the postoperative PCO had the greatest impact on the final ROM [14]. There was significant correlation between the postoperative PCO and ROM at 12 months after surgery (r = 0.65; *p* < 0.0001). However, the relationship between the postoperative PCO and postoperative ROM was not consistently reproduced in the following studies using PCL-substituting or cruciate-sacrificing implants [12, 13]. In the present study, there was no significant correlation between differences in the PCO or PCOR and postoperative ROM, regardless of referencing systems used. The contradictory findings of our study to those of *Bellemans et al*. may be explained in part by the fact that we used a different design of the PCL-substituting implant.

*Alexander et al*. demonstrated that there are no statistically significant differences in surgical or clinical outcomes between the AR and PR systems. The mean Knee Society Score at 2 years after TKA was 98.11 in the AR group and 97.69 in the PR group (*p* = 0.7647, 5). In our study, the magnitude of improvement in WOMAC score after surgery showed no difference between the two groups. On the other hand, both the mean postoperative ROM and improvement of ROM were greater in AR group. However, both groups achieved a mean postoperative ROM greater than 120°. If a flexion arc of 110° or more is achieved, improvement of activities in daily life can be expected [23]. Combining the findings of previous studies and this study, the AR and PR systems would both contribute to the comparable clinical outcomes after TKA using a PCL-substituting fixed-bearing implant.

This study has several limitations. First, the study was retrospectively performed and AR and PR group depends on man’s preference, this can lead to selection bias.. Second, a true lateral radiograph was used to assess the PCO and PCOR, which cannot reflect cartilage thickness. However, all pre- and post-TKA radiographic parameters were measured twice, and intra- and inter-observer measurement reliabilities showed good to excellent agreement. This suggests that the PCO and PCOR values used in this study were reliable. Third, the results may not be generalized to all TKA patients because of the various implant designs. However, this study provides valuable information to readers by comparing two referencing systems in the same PCL-substituting fixed-bearing implant. Fourth, The evaluation of the condyles of the femur on plain lateral X-ray has limitations on evaluating each medial and lateral condyle of femur accurately. However, the accurate evaluation by CT is also difficult due to the influence of the metal artifact. Moreover, in many previous studies, plain X-ray was mainly used for evaluation, so it was easier to compare with other studies. Also, Evaluating patients with CT for the study is not ethical. So we did research using X-ray. Finally, the follow-up period of two years was relatively short. However, the two-year follow-up was enough to present postoperative ROM and knee scores because the postoperative ROM rarely changes one year after surgery [24].

## Conclusions

There was no difference in postoperative PCO in AR and PR group and the PCO was more consistently preserved after surgery in the PR group. The postoperative PCO and PCOR changes did not affect the postoperative ROM, regardless of the referencing system used after PCL-substituting fixed-bearing TKA. Furthermore, comparable satisfactory clinical outcomes were achieved in both groups.

## Data Availability

The datasets used or analysed during the current study are available from the corresponding author on reasonable request.

## Abbreviations

AR: Anterior referencing system
PCO: Posterior condylar offset
PCOR: Posterior condylar offset ratio
PR: Posterior referencing system
